# Core Minimal Datasets to Advance Clinical Research for Priority Epidemic Diseases

**DOI:** 10.1093/cid/ciz760

**Published:** 2019-08-13

**Authors:** Amanda M Rojek, James Moran, Peter W Horby

**Affiliations:** Epidemic Diseases Research Group, University of Oxford, United Kingdom

**Keywords:** Ebola virus disease, epidemic, pandemic, emerging infection

## Abstract

The Ebola virus disease outbreak in west Africa has prompted significant progress in responding to the clinical needs of patients affected by emerging infectious disease outbreaks. Among the noteworthy successes of vaccine trials, and the commendable efforts to implement clinical treatment trials during Ebola outbreaks, we should also focus on strengthening the collection and curation of epidemiological and observational data that can improve the conception and design of clinical research.

## Teaser

There is an urgent need to improve observational data collection during emerging infectious disease outbreaks. Clinical treatment trials and public health decision making will benefit from curation of high-quality clinical characterization and epidemiological data.

During the currently ongoing Ebola virus disease (EVD) outbreak in the Democratic Republic of Congo, a clinical trial of potential treatments has commenced. This is a significant step toward improving outcomes for patients with the disease.

Ebola virus disease constitutes but one of the priority diseases that the World Health Organization (WHO), in their Blueprint for Action to Prevent Epidemics, suggests poses a severe public health risk and for which there are insufficient countermeasures [1]. The purpose of this priority list is to identify high-threat pathogens for which there is a need to prioritize and advance the development of diagnostics, vaccines, and therapeutics. Any diagnostics, drugs, or vaccines that are developed as a result of this and other initiatives, such as the Coalition for Epidemic Preparedness Innovation, will need to be fully evaluated in diagnostic evaluation studies or phase II and III clinical trials.

However, due to the very nature of the epidemic-prone infectious diseases that appear in the WHO list of priority diseases, evaluation in clinical studies is challenging, not least because the epidemiology is unpredictable but also because the pathogenesis and natural history of many of these diseases are not well defined. For example, during the influenza A(H1N1)pdm09 pandemic, case fatality rate (CFR) estimates varied widely from 0 to 13 500 per 100 000 laboratory-confirmed infections, with a heterogeneity of 99.97% (using *I*^2^ estimate) [2]. A therapeutic trial designed with patient survival as a primary outcome measure would have grossly misjudged the required sample size if the trial was designed using the wrong CFR. Therapeutic trials for the prevention of congenital Zika syndrome will be hindered by the absence of consistently used criteria to define the outcome of congenital malformations [3]. For Middle East respiratory syndrome coronavirus, a lack of systematic biological sampling means that disease pathophysiology and factors associated with more severe disease and viral clearance (a commonly used secondary outcome measure) are not well understood [4].

The need for well-defined core minimal datasets for emerging infectious diseases is not a new observation. A decade ago Sheila Bird and Jeremy Farrar [5] noted the need to define a core minimal dataset for human cases of avian influenza A/H5N1, yet there remains no systematic examination of the completeness of the core data needed to design and conduct trials for high-priority pathogens. Table 1 identifies some key domains that could contribute to a core minimal dataset that informs clinical trial design for each priority pathogen.

**Table 1.** Suggested Elements for a Core Minimal Dataset of Observation-based Data for Designing Clinical Trials for High-priority Pathogens

Nature of Information	Value to the Conception and Design of Clinical Trials
Case counts for previous outbreaks	Serves as rudimentary estimate of the feasibility of sample-size requirements. Clinical trial groups should prioritize the most efficient trial designs when a low number of cases is expected.
Temporal and geographical profile of previous outbreaks	This is required for logistical planning, to ensure that local teams are sufficiently trained in research practices (such as good clinical practice) and trial-specific equipment is available.
An agreed-upon case definition	Clinical characteristics of the disease are used to define enrollment criteria.
Analysis of strength of evidence for factors associated with increased disease severity or fatality	Stratification (or other statistical adjustment) on the basis of severity is often required when interpreting the clinical trial outcome.
Best available descriptions of the type and rate of clinical outcomes	Clinical outcomes will function as a trial outcome measures. Understanding the natural course of illness will also help differentiate disease course from adverse events from treatment.
Assessment of confidence in estimates of clinical outcomes	Heterogeneity in patient outcomes between or within outbreaks creates uncertainty for power calculations and will affect selection of a statistical design for a trial. Spurious heterogeneity may occur due to random error in small cohorts, or represent ascertainment, lead-time, measurement, or follow-up bias. Real heterogeneity can occur due to improvements in care over an outbreak, pathogen evolution, or changes in host susceptibility and vulnerability but should be adjusted for.
Analysis of known or suspected covariates of outcome	Highlights possible confounders that will alter outcome independently of treatment and that will require adjustment if unequally distributed between treatment and control arms.
The mean time from onset of symptoms to outcome	Allows for an estimation of the feasibility and logistics of medical intervention.
Agreed-upon standards of care for patient treatment	Determines if there is standardized supportive therapy to be adopted in all arms of a trial. This is especially important for multicenter research
The performance characteristics of the favored diagnostic method	Determines whether a trial will be performed on an ITT basis or following laboratory confirmation.
Mean time for laboratory diagnosis	Determines whether a trial will be performed on an ITT basis or following laboratory confirmation.
Community priorities and expectations for trials	Determines the priorities of affected communities in terms of access to trials, acceptable methodology, and acceptability of treatments or vaccines.

Abbreviation: ITT, intention-to-treat.

The benefit of this approach, when complemented by scoring or assessment of the available information, is that it allows for initial bench-marking and triaging of unmet data needs in order to prioritize further data gathering activities. Importantly, a harmonized data collection initiative can also prospectively embed data-sharing agreements into data-collection protocols. This will allow valuable clinical information to be readily available to stakeholders, while identifying and protecting the interests of those collecting data in regions where outbreaks occur.

Accumulation and curation of the data will depend on a variety of sources and methodology types, but it is critical that high-quality clinical data are highlighted as an integral component. Often lost to competing priorities for clinicians during outbreaks, standardized data collection regarding the presentation and natural history of disease, biomarkers of disease severity, and response to supportive care can be sporadic or missing. While these data have their most important benefits in improving patient management (through better recognition of disease complications and informing supportive care) and public health control, patient-based data are also used to determine key parameters for clinical trials, such as the inclusion criteria, the nature and rate of clinically relevant outcomes, and potential confounders. We suggest that adoption of clinical case registries (such as those used for rare cancers) provides a feasible option to produce standardized clinical data that have multiple clinical, public health, and research benefits [6].

Compared with expensive and lengthy countermeasure development pipelines, improving the scale, relevance, and quality of observational data is likely to be an efficient and cost-effective strategy to improve global preparedness against epidemic and pandemic infections.

## Notes

***Authors contributions.*** A. M. R., J. M., and P. W. H. conceived of the manuscript, contributed to drafting, and agree with the contents.

***Disclaimer.*** The funders had no role in the study design; in the collection, analysis, and interpretation of data; in the writing of the report; or in the decision to submit the paper for publication.

***Financial support.*** This work was supported by the Wellcome Trust of Great Britain (grant numbers 107834/Z/15/Z and 106491/Z/14/Z). A. M. R. was funded by a Rhodes Scholarship.

***Potential conflicts of interest.*** The authors report no potential conflicts of interest. All authors have submitted the ICMJE Form for Disclosure of Potential Conflicts of Interest. Conflicts that the editors consider relevant to the content of the manuscript have been disclosed.
